# Venezuelan equine encephalitis virus infection causes modulation of inflammatory and immune response genes in mouse brain

**DOI:** 10.1186/1471-2164-9-289

**Published:** 2008-06-16

**Authors:** Anuj Sharma, Bhaskar Bhattacharya, Raj K Puri, Radha K Maheshwari

**Affiliations:** 1 Centre for Combat Casualty and Life Sustainment Research, Department of Pathology, Uniformed Services University of the Health Sciences, Bethesda, Maryland, USA; 2 Biological Sciences Group, Birla Institute of Technology and Science, Pilani, India; 3 Tumor Vaccines and Biotechnology Branch, Division of Cellular and Gene Therapies, Center for Biologics Evaluation and Research, Food and Drug Administration, Bethesda, Maryland, USA

## Abstract

**Background:**

Neurovirulent Venezuelan equine encephalitis virus (VEEV) causes lethal encephalitis in equines and is transmitted to humans by mosquitoes. VEEV is highly infectious when transmitted by aerosol and has been developed as a bio-warfare agent, making it an important pathogen to study from a military and civilian standpoint. Molecular mechanisms of VEE pathogenesis are poorly understood. To study these, the gene expression profile of VEEV infected mouse brains was investigated. Changes in gene expression were correlated with histological changes in the brain. In addition, a molecular framework of changes in gene expression associated with progression of the disease was studied.

**Results:**

Our results demonstrate that genes related to important immune pathways such as antigen presentation, inflammation, apoptosis and response to virus (*Cxcl10*, *CxCl11*, *Ccl5*, *Ifr7*, *Ifi27 Oas1b*, *Fcerg1*,*Mif*, *Clusterin and MHC class II) *were upregulated as a result of virus infection. The number of over-expressed genes (>1.5-fold level) increased as the disease progressed (from 197, 296, 400, to 1086 at 24, 48, 72 and 96 hours post infection, respectively).

**Conclusion:**

Identification of differentially expressed genes in brain will help in the understanding of VEEV-induced pathogenesis and selection of biomarkers for diagnosis and targeted therapy of VEEV-induced neurodegeneration.

## Background

Venezuelan equine encephalitis virus (VEEV) is an alphavirus in the family *Togaviridae*. It causes a highly virulent central nervous system (CNS) disease in horses and other equines and is transmitted to humans by mosquitoes. Outbreaks of VEEV have been reported at intervals of 2 to 10 years in South and Central America and in the Texas region of North America, which has resulted in VEEV being included on the list of emerging pathogens [1]. VEEV has also been developed as a bio-warfare agent, making its use more likely than other non-weaponized agents in the event that a bio-weapon or bio-terrorism agent is used [2]. There is no specific treatment available for VEEV. A vaccine for prophylaxis against VEEV is under investigational new drug status and is given to personnel at risk of occupational exposure to VEEV. This vaccine has several limitations and has not been licensed by the FDA. VEEV infection in mice induces the bi-phasic disease observed in equines and lethal human infections (~0.5%) thus making it a good model to study VEEV pathogenesis [3,4]. VEEV spreads from the site of inoculation through the locally draining lymph nodes, causes viremia, and disseminates to other lymphoid organs [5]. Viremia is followed by a CNS phase of disease [6-8]. VEEV enters into the CNS primarily through the olfactory neuroepithelium, via brain capillary endothelial cells and the trigeminal nerve [7,9]. In the CNS, VEEV infects neurons and glial cells and causes subsequent cellular degeneration. Infection with VEEV results in neuronal cell death, active gliosis and an intense inflammatory response characterized by perivascular and interstitial mononuclear cell infiltration [4,5,10-13]. However, in another subset of dying neurons associated with astrogliosis, no VEEV antigen can be found, indicating an alternate, indirect mechanism of neuronal degeneration [11,13].

As the mechanism(s) underlying the inflammatory and immune response to VEEV infection in brain and subsequent neurodegenration are poorly understood, it is important to characterize the underlying cellular and molecular pathways. To fulfill this objective we used the virulent neuroinvasive strain, V3000, of VEEV. Gene expression changes in VEEV-infected mouse brain were studied using microarrays containing 16,463 oligonucleotides, representing 15,000 genes. We demonstrate that with the progression of disease both the total number of genes and fold expression of numerous genes increased. Many of the changes were associated with important immune pathways such as antigen presentation, inflammation, apoptosis and viral response. Gene expression changes were validated by RT-PCR. Pathologic changes in the brain were documented by H&E (hematoxylin and eosin) and immunohistochemistry (IHC).

## Results

### Survival of animals post VEEV infection

Mice began showing roughening of fur and hunched back at day 2 post infection. By day 4, animals were lethargic and at least one mouse had hind limb paralysis. One animal died on day 5 post infection and all other animals showed little or no movement. All the mice died by day 9 post infection. Mortality was 100% with mean survival time of 6.6 days. Animal mortality is documented in Table 1.

**Table 1:** Progression of disease and mortality in VEEV infected mice.

Disease Progression	Day(s) Post Infection									
	0	1	2	3	4	5	6	7	8	9
Roughening of Fur	0	0	5	9	9	8	5	1	1	-
Hunched Posture/Lethargic/Shivering	0	0	0	0	9	8	5	1	1	-
Hind Limb Paralysis	0	0	0	0	1	8	5	1	1	-
Death	0	0	0	0	0	1	3	4	0	1
Total died/Total No.	0/9	0/9	0/9	0/9	0/9	1/9	4/9	8/9	8/9	9/9
% Mortality	0	0	0	0	0	11.1	44.4	88.8	88.8	100
MST (Day)	6.66									

Mice were infected with 1000 pfu of V3000 in left rear foot pad at day 0 and were monitored for the clinical symptoms of disease thereafter. All the animals exhibited hind limb paralysis, a hallmark of VEEV infection and succumbed to disease by day 9 post infection. Mortality was 100% with a mean survival time of 6.6 days.

### Histopathology and immunohistochemistry for VEEV antigen

**Figure 1:**
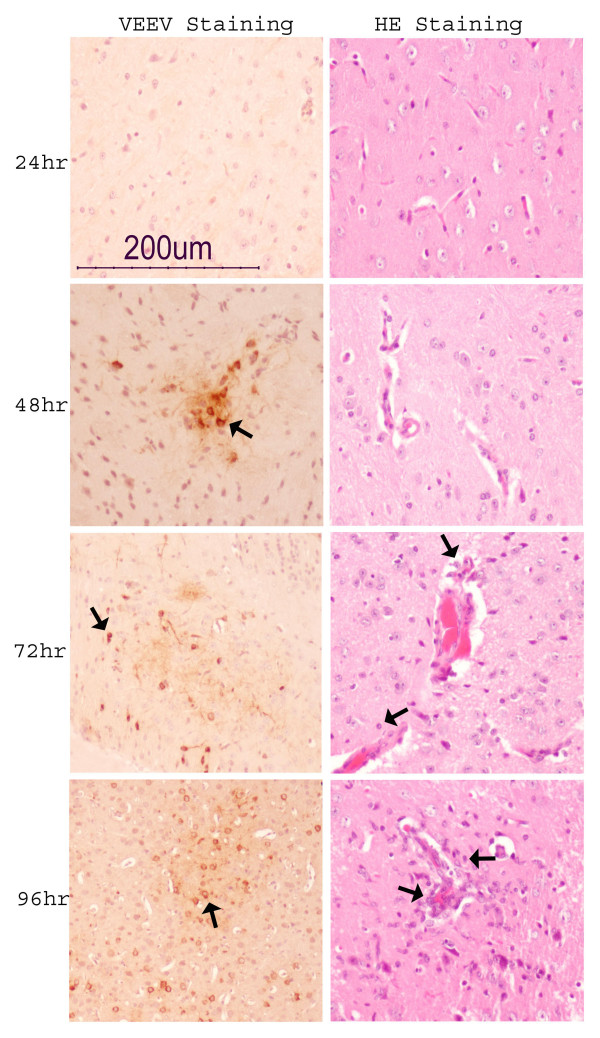
**Evidence of VEEV infection in mouse brain**. Mouse brains at various time points after VEEV infection were analyzed by H&E and IHC staining. VEEV appeared in brain at 48 hr post infection (indicated by arrows), which was accompanied by the initiation of inflammation in brain as evident by vessel thickening and endothelial cuffing. At 72 hr post infection VEEV antigen can be detected throughout the mid-brain. Inflammation was evidenced by increased endothelial cuffing and neutrophil infiltration (indicated by arrows). Inflammation was extensive throughout the brain along with increased VEEV antigen in brain at 96 hr post infection.

VEEV antigen-specific staining showed the presence of viral antigen within neurons and glial cells in the olfactory and prefrontal areas of the brain at 48 hr post infection. Many more VEEV infected neurons and glial cells were present throughout the brain at 72 hr post infection. VEEV antigen was present throughout the brain at 96 hr post infection and the degree of inflammation corresponded to the density of virus infected cells. Inflammation started with perivascular cuffing and localizaton to the meninges at 48 hr post infection. At 72 hr post infection, inflammation was observed in the olfactory and frontal area of the brain. By 96 hr post-infection inflammation was extensive and had spread throughout the brain (Fig 1).

### Gene expression analysis

Microarrays were performed using RNA extracted from VEEV-infected mouse brain at 24 hr, 48 hr, 72 hr and 96 hr post infection and gene expression profile were compared with RNA extracted from saline infected control mice at similar time points. All the biological replicates (duplicates for each time point) shared significant homology in gene expression (correlation coefficient ≥ 0.80). Mean fold-expression of biological replicates ≥ 1.5 fold over control was determined to be significant. As shown in Table 2, the number of genes upregulated in brain increased as disease progressed (Fig 1). At 96 hr post infection the maximum numbers of total genes were upregulated at ≥ 1.5 fold levels in mouse brain.

**Table 2:** Total Genes Upregulated in VEEV Infected Mice brain.

Time, Hour(s) pi	> 5.0 folds	> 3.0–5.0 folds	≥ 1.5–3.0 folds	Total genes over-expressed
24	6	18	183	207
48	3	27	292	322
72	53	148	212	413
96	33	129	960	1122

Total RNA extracted from VEEV infected brain was analyzed by microarray as described in methods. Gene's upregulated ≥1.5 folds were considered. Number of genes over expressed increased as the disease progressed with maximum genes over expressed at 96 hr post infection.

**Figure 2:**
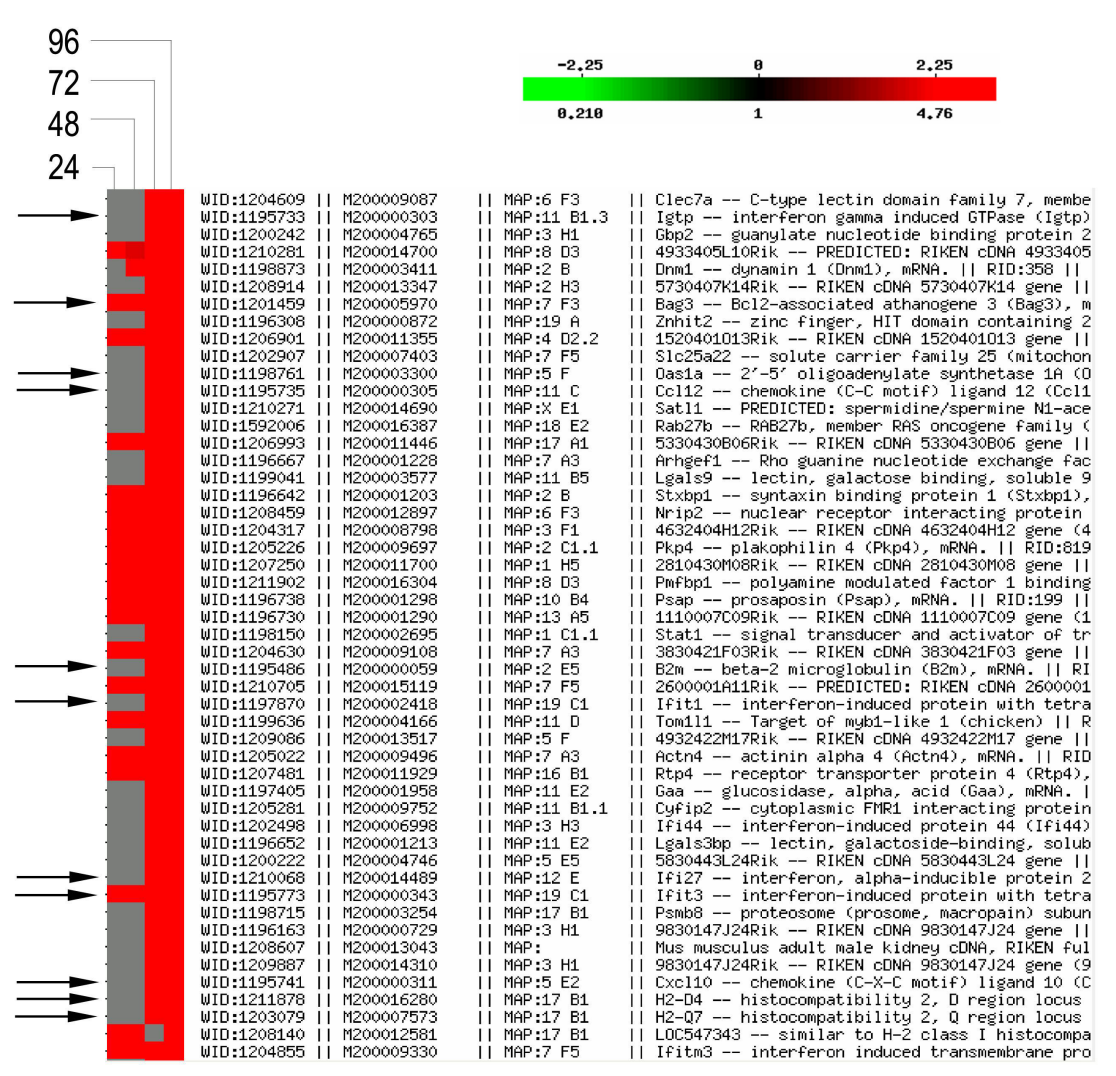
**Hierarchical clustering of gene expression in mouse brain after VEEV infection**. All over-expressed genes were clustered; however, a snap shot of selected genes is shown due to space limitation. Genes that are marked with arrows are over expressed ≥ 2.0 fold at 72 and 96 hr post infection. Color indicates the relative expression level of each gene in VEEV infected brain over saline treated control mouse brain, with red indicating higher expression, grey indicating absence of expression and darker red color indicating over expression but at a lower level.

To analyze similarities and difference in gene expression between brains at different time points, hierarchical clustering analysis was done for all genes at the four time points. Genes which showed a distinct pattern of over-expression with the time of VEEV infection are shown in Fig 2. All genes were grouped in two clusters. The first cluster contained genes upregulated at 24 hr and 48 hr, whereas the second cluster contained genes upregulated at 72 hr and 96 hr post infection. This analysis clearly distinguished the changes in the gene expression profile with the progression of VEEV infection. Some genes related to the inflammatory response, such as *Cxcl10, Clu *and antigen presentation, such as *B2m*, *Fcer1g *showed over-expression only at 72 and 96 hr post infection (Fig 2).

**Figure 3:**
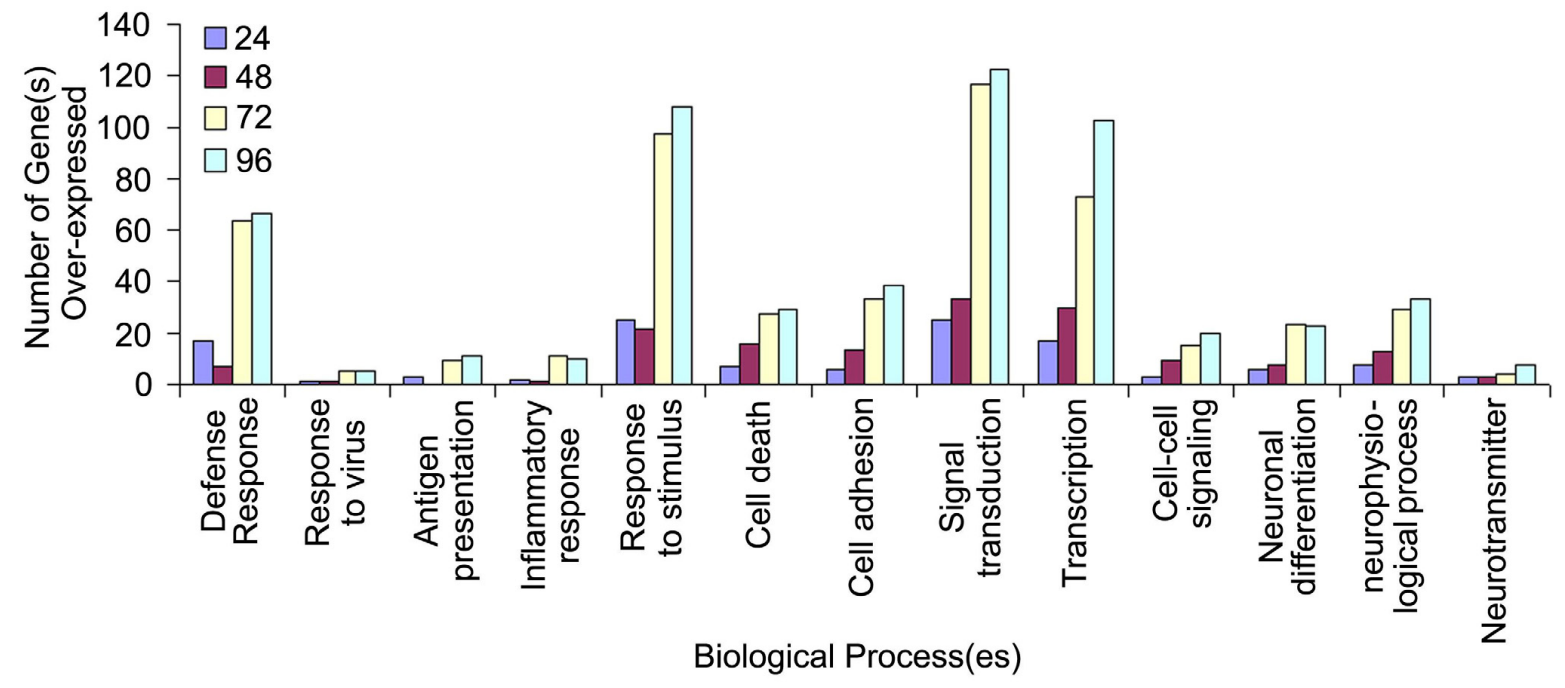
**Analysis of molecular pathways in VEEV infected brain**. Number of genes involved in various pathways of biological processes was analyzed by GOFFA Library of Arraytrack software. Genes involved in the various biological processes increased with the progress of the disease.

Functional analysis of over-expressed genes was performed using the GOFFA (gene ontology for functional analysis) library of the Arraytrack software. Genes belonging to various biological processes were grouped together. Based on these analyses, a majority of genes (164, 251, 759, 928) were identified to play a role in different biological processes at 24 hr, 48 hr, 72 hr, and 96 hr post infection, respectively. These genes were further categorized by the various functions that may play an important role in VEEV pathogenesis (Fig 3) as follows:

#### i) Virus response genes

It has been shown that interferon and interferon-inducible genes play an important role in the immune response towards viral pathogens. Consistent with this information, several interferon-inducible genes were upregulated (Table 3) in present study. These include *Ifi27*, *Ifih1*, *Irf7 *and *Oas1b *which were upregulated at 72 hr and 96 hr post infection except *Becn1 *that was over-expressed only at 72 hr post infection, and *Spn *which was over-expressed at all time points.

**Table 3:** Genes involved in virus response, inflammatory response and antigen presentation

**UniGene ID**	**Functional Grouping (Gene)**	**Upregulation (fold change) (Hours post infection)**			
		
		**24**	**48**	**72**	**96**
	**Virus Response and Inflammation**				

Mm.136224	Interferon induced with helicase C domain 1 (Ifih1)	NE	3.59/-	5.21 ± 1.05	4.18 ± 0.31
Mm.3233	Interferon regulatory factor 7 (Irf7)	NE	NE	4.31 ± 1.51	2.01 ± 0.08
Mm.271275	Interferon, alpha-inducible protein 27 (Ifi27)	NE	NE	4.79 ± 0.67	6.62 ± 0.32
Mm.233471	2'-5' oligoadenylate synthetase 1B (Oas1b)	NE	1.89/-	5.12 ± 0.04	2.32 ± 0.08
Mm.867	Chemokine (C-C motif) ligand 12 (Ccl12)	5.03 ± 1.86	4.63/-	7.87 ± 0.54	4.70 ± 0.36
Mm.131723	Chemokine (C-X-C motif) ligand 11 (Cxcl11)	NE	8.76/-	17.02 ± 5.19	13.68 ± 5.51
Mm.877	Chemokine (C-X-C motif) ligand 10 (Cxcl10)	NE	7.49/-	15.45 ± 0.67	8.69 ± 0.37
Mm.284248	Chemokine (C-C motif) ligand 5 (Ccl5)	NE	NE	4.27 ± 1.27	3.56 ± 0.66
Mm.1282	Chemokine (C-C motif) ligand 3 (Ccl3)	NE	NE	2.26/1.83	2.05 ± 0.21
Mm.10116	Chemokine (C-X-C motif) ligand 13 (Cxcl13)	NE	NE	2.06 ± 0.41	1.73/-
Mm.766	Chemokine (C-X-C motif) ligand 9 (Cxcl9)	NE	NE	3.99 ± 1.62	1.85 ± 0.14
Mm.22673	Fc receptor, IgE, high affinity I, gamma polypeptide (Fcer1g)	NE	NE	3.66 ± 0.29	3.38 ± 0.63
Mm.5419	Interleukin 17 (Il17)	NE	NE	NE	2.06 ± 0.33
Mm.2326	Macrophage migration inhibitory factor (Mif)	NE	1.08 ± 0.17	2.25 ± 0.53	2.06 ± 0.07
Mm.24163	PYD and CARD domain containing (Pycard)	NE	NE	1.81 ± 0.165	1.52 ± 0.31
Mm.200608	Clusterin (Clu)	NE	1.74 ± 0.13	4.03 ± 1.54	2.45 ± 0.37
Mm.283714	Sialophorin (Spn)	1.93 ± 0.14	2.50 ± 0.19	2.87 ± 1.63	2.25 ± 0.24

	**Antigen Presentation**				

Mm.209294	Adaptor-related protein complex 3, delta 1 subunit (Ap3d1)	1.33 ± 0.83	NE	2.17 ± 0.35	1.69 ± 0.01
Mm.163	Beta-2 microglobulin (B2m)	NE	NE	10.39 ± 0.32	6.07 ± 0.25
Mm.1894	CD1d1 antigen (Cd1d1)	NE	NE	NE	1.56 ± 0.01
Mm.22673	Fc receptor, IgE, high affinity I, gamma polypeptide (Fcer1g)	NE	NE	3.66 ± 0.29	3.38 ± 0.63
Mm.33263	Histocompatibility 2, D region locus 1 (H2-D1)	NE	NE	4.88 ± 0.12	9.04 ± 1.15
Mm.387141	Histocompatibility 2, D region locus 4 (H2-D4)	NE	NE	8.92 ± 7.66	9.42 ± 1.34
Mm.35016	Histocompatibility 2, T region locus 23 (H2-T23)	NE	NE	4.72 ± 0.89	3.15 ± 0.83
Mm.296901	Histocompatibility 2, Q region locus 7 (H2-Q7)	4.55 ± 2.49	4.58/-	10.22 ± 4.92	9.50 ± 0.76
Mm.296901	Histocompatibility 2, Q region locus 8 (H2-Q8)	NE	NE	4.45 ± 0.48	NE

Genes that are involved in the virus response and immune response were upregulated at 72 and 96 hr post infection concomitant with the virus appearance in the brain. Several inflammatory response and antigen presentation genes were upregulated and their expression increased with the progression of the disease. Values are fold changes in the gene expression in test samples over saline controls. Values are expressed as mean ± SEM. NE indicates not expressed or expression was lower than 1 fold (and/or did not meet the cut off criteria of 99% confidence interval, 150 minimum intensity or 30 μm spot size) NE indicates not expressed and '-' indicates not expressed in biological replicates.

#### ii) Inflammatory response genes

Inflammation constitutes an important part of the immune response towards the invading pathogen. In VEEV infection, inflammation in brain is implicated in the secondary neuronal damage leading to morbidity and mortality. Several genes related to the inflammatory response were upregulated in the brain following VEEV infection, mostly at 72 and 96 hr post infection (Table 3). Most importantly, chemokine genes *e.g., Cxcl9, Cxcl10, Cxcl11, Cxcl13*, *Ccl3, Ccl5 *and *Ccl12 *that exert a chemotactic signal for the immune cell migration to the site of the injury were upregulated at 72 and 96 hr post infection. These gene expression correlates with the increased neutrophil infiltration in brain and blood brain barrier (BBB) compromise (Fig 1). In addition inflammatory genes *e.g., Fcer1g*, and *Mif *were also over-expressed at 72 and 96 hr post infection. (Table 3)

#### iii) Genes involved in antigen presentation (AP)

Resident glial cells of the brain are known to act as antigen presenting cells and constitute an important part of the immune response against a virus pathogen. Several major histocompatibility complex (MHC) class II genes *e.g., H2-D1, H2-D4 H2-Q7 and H2-T23 *were upregulated at 72 hr and 96 hr post infection, concomitant with VEEV antigen appearance in the brain. MHC class I receptors such as *Cd1d1*, *B2m *and *Ap3d1 *were also upregulated at 96 hr post infection. (Table 3)

#### iv) Apoptotic gene expression

Apoptotic neurons were seen in the VEEV-infected mouse brain in regions associated with VEEV antigen and the regions of gliosis that are free of VEEV antigen. Several apoptotic genes such as caspase recruitment domain (*Card*)14, fas apoptotic inhibitory molecule (*Faim*)2, Apoptosis-associated tyrosine kinase (*Aatk*), eukaryotic translation initiation factor 5 (*Eif5a*) and myeloid cell leukemia sequence 1 (*Mcl1*) were upregulated at 72 hr and 96 hr post infection (Fig 3). Few genes, like amyloid beta (A4) precursor protein (*App*), bcl2/adenovirus e1b interacting protein 3-like (*Bnip3l*), baculoviral IAP repeat-containing 6 (*Birc6*) and *Spn *were upregulated throughout the study and few others such as amyloid beta (*A4*) precursor-like protein 1 (*Aplp1*), Clusterin *(Clu)*, integral membrane protein 2B (*Itm2b)*, and valosin containing protein (*Vcp*), were upregulated as early as 48 hr post infection (Table 4).

**Table 4:** Genes involved in apoptotic response in VEEV infected mice brain.

**UniGene ID**	**Functional Grouping (Gene)**	**Upregulation (fold change) (Hours post infection)**			
		
		**24**	**48**	**72**	**96**
	**Apoptosis**				

Mm.3336	RIKEN cDNA 1110007C09 gene (1110007C09Rik)	2.66 ± 1.63	3.68 ± 1.14	6.81 ± 4.35	5.22 ± 0.66
Mm.277585	amyloid beta (A4) precursor protein (App)	2.50 ± 0.48	2.54 ± 0.59	4.05 ± 1.20	2.87 ± 0.42
Mm.84073	Bcl2-associated athanogene 3 (Bag3)	2.63 ± 0.59	3.30 ± 0.43	4.61 ± 2.37	4.23 ± 0.18
Mm.29820	BCL2/adenovirus E1B 19kDa-interacting protein 3-like (Bnip3l)	1.96 ± 0.11	2.52 ± 0.04	3.88 ± 2.19	3.07 ± 0.14
Mm.290908	baculoviral IAP repeat-containing 6 (Birc6), mRNA.	1.94 ± 0.33	2.15 ± 0.22	2.54 ± 0.93	2.18 ± 0.04
Mm.283714	sialophorin (Spn), mRNA.	1.99 ± 0.14	2.50 ± 0.19	2.87 ± 1.63	2.25 ± 0.24
Mm.2381	amyloid beta (A4) precursor-like protein 1 (Aplp1),	NE	1.90 ± 0.06	3.70 ± 1.15	2.27 ± 0.14
Mm.200608	Clusterin (Clu)	NE	1.74 ± 0.13	4.03 ± 1.54	2.45 ± 0.37
Mm.136224	interferon induced with helicase C domain 1 (Ifih1)	NE	3.59/-	5.21 ± 1.05	4.18 ± 0.31
Mm.4266	integral membrane protein 2B (Itm2b)	NE	1.55 ± 0.20	2.43 ± 0.95	2.46 ± 0.54
Mm.2326	macrophage migration inhibitory factor (Mif)	NE	1.08 ± 0.17	2.25 ± 0.53	2.06 ± 0.07
Mm.277518	Monocyte to macrophage differentiation-associated (Mmd)	NE	0.59 ± 0.04	2.33 ± 0.45	1.64 ± 0.18
Mm.347546	Inositol 1,3,4-triphosphate 5/6 kinase	NE	1.94 ± 0.02	0.95 ± 0.05	1.83 ± 0.02
Mm.285322	mitochondrial carrier homolog 1 (C. elegans) (Mtch1)	NE	0.95 ± 0.01	2.08 ± 0.35	1.77 ± 0.23
Mm.687	Ras homolog gene family, member B (Rhob)	NE	1.17 ± 0.03	2.48 ± 0.87	1.59 ± 0.15
Mm.379457	Valosin containing protein (Vcp)	NE	1.68 ± 0.15	2.04 ± 0.77	1.80 ± 0.17
Mm.6826	Apoptosis-associated tyrosine kinase (Aatk)	NE	NE	1.65 ± 0.07	1.20 ± 0.43
Mm.223689	Bifunctional apoptosis regulator (Bfar)	NE	NE	1.83 ± 0.58	1.70 ± 0.08
Mm.130832	caspase recruitment domain family, member 14 (Card14)	NE	NE	3.23 ± 1.52	2.69 ± 0.08
Mm.29028	Death associated protein 3 (Dap3)	NE	NE	1.87 ± 0.14	0.96 ± 0.03
Mm.280594	Death effector domain-containing DNA binding protein 2 (Dedd2)	NE	NE	1.79 ± 0.66	1.92 ± 0.09
Mm.379461	eukaryotic translation initiation factor 5A (Eif5a)	NE	NE	2.61 ± 0.49	1.92 ± 0.14
Mm.342392	engulfment and cell motility 1, ced-12 homolog (C. elegans) (Elmo1), transcript variant 2	NE	NE	1.74 ± 0.21	1.41 ± 0.08
Mm.39760	Fas apoptotic inhibitory molecule 2 (Faim2)	NE	NE	2.85 ± 1.23	2.44 ± 0.12
Mm.281298	growth arrest and DNA-damage-inducible 45 gamma (Gadd45g)	NE	NE	3.16 ± 0.21	2.12 ± 0.05
Mm.15510	granzyme A (Gzma)	NE	NE	1.27 ± 0.41	1.94 ± 0.26
Mm.2720	Mitogen activated protein kinase 8 interacting protein 1 (Mapk8ip1)	NE	NE	2.25 ± 0.75	1.16 ± 0.19
Mm.1639	Myeloid cell leukemia sequence 1 (Mcl1)	NE	NE	1.83 ± 0.18	1.56 ± 0.09
Mm.204876	nucleolar protein 3 (apoptosis repressor with CARD domain) (Nol3)	NE	NE	2.02 ± 0.52	1.84 ± 0.09
Mm.24163	PYD and CARD domain containing (Pycard)	NE	NE	1.82 ± 0.17	1.38 ± 0.18
Mm.276325	superoxide dismutase 1, soluble (Sod1)	NE	NE	1.74 ± 0.22	1.54 ± 0.24
Mm.200792	tumor necrosis factor receptor superfamily, member 21 (Tnfrsf21)	NE	NE	2.37 ± 0.84	1.40 ± 0.17
Mm.1894	CD1d1 antigen (Cd1d1)	NE	NE	NE	1.56 ± 0.01
Mm.217764	phosphoglucomutase 2 (Pgm2)/Itgb3bp	NE	NE	NE	1.63 ± 0.11
Mm.312628	Serine (or cysteine) proteinase inhibitor, clade A, member 3G (Serpina3g)	NE	NE	NE	2.83 ± 0.18
Mm.218473	tumor differentially expressed 1 (Tde1)/Serinc3	NE	NE	NE	1.94 ± 0.01
Mm.338613	forkhead box O3a (Foxo3a)	1.92 ± 0.17	NE	2.26 ± 1.08	1.57 ± 0.03
Mm.22216	TSC22 domain family 3 (Tcs22d3)	NE	1.63 ± 0.20	NE	NE
Mm.1360	growth arrest and DNA-damage-inducible 45 beta (Gadd45b)	NE	2.00 ± 0.33	NE	1.85 ± 0.03
Mm.329277	ubiquitin specific peptidase 14 (Usp14)/Thoc1	NE	1.90 ± 0.21	NE	2.02 ± 0.18
Mm.347406	CCAAT/enhancer binding protein (C/EBP), beta (Cebpb)	NE	NE	2.01 ± 0.17	NE
Mm.150	Fc receptor, IgG, high affinity 1(Fcgr1)	NE	NE	2.26 ± 0.26	NE
Mm.292100	fibrinogen-like protein 2 (Fgl2)	NE	NE	1.90 ± 0.27	NE
Mm.368515	myc-like oncogene, s-myc protein (Mycs)	NE	NE	3.11 ± 0.05	NE

Genes that are modulated in the apoptotic response were upregulated at 72 and 96 hr post infection concomitant with the virus appearance in the brain. Values are fold changes in the gene expression in test samples over saline controls. Values are expressed as mean ± SEM. NE indicates not expressed or expression was lower than 1 fold (and/or didn't meet the cut off criteria of 99% confidence interval, 150 minimum intensity or 30 μm spot size) and (-) indicates not expressed in biological replicates.

Apart from over-expressed genes, few genes were down-regulated (≤2 fold) at all time points. Those genes are listed in supplementary table 1. As expected, the functional analysis showed genes related to several cellular physiological processes such as cell transport (*Atp5j2, Slc18a2, Actr6, Timm8b*) cell cycle/division (*Cetn3*), cellular metabolism (*Zfp99, Rpl37a, Pigf, Snrpe, Rps26, Rps12, Rpl39, Rpl26, Rpl30, Cox7c, Ndufc1, Mrps33 *and *Ppia*) and other cellular processes (*stmn3, Pvalb, Pcp4, Usmg5*) were down-regulated in VEEV infection.

### Confirmation of gene expression

**Figure 4:**
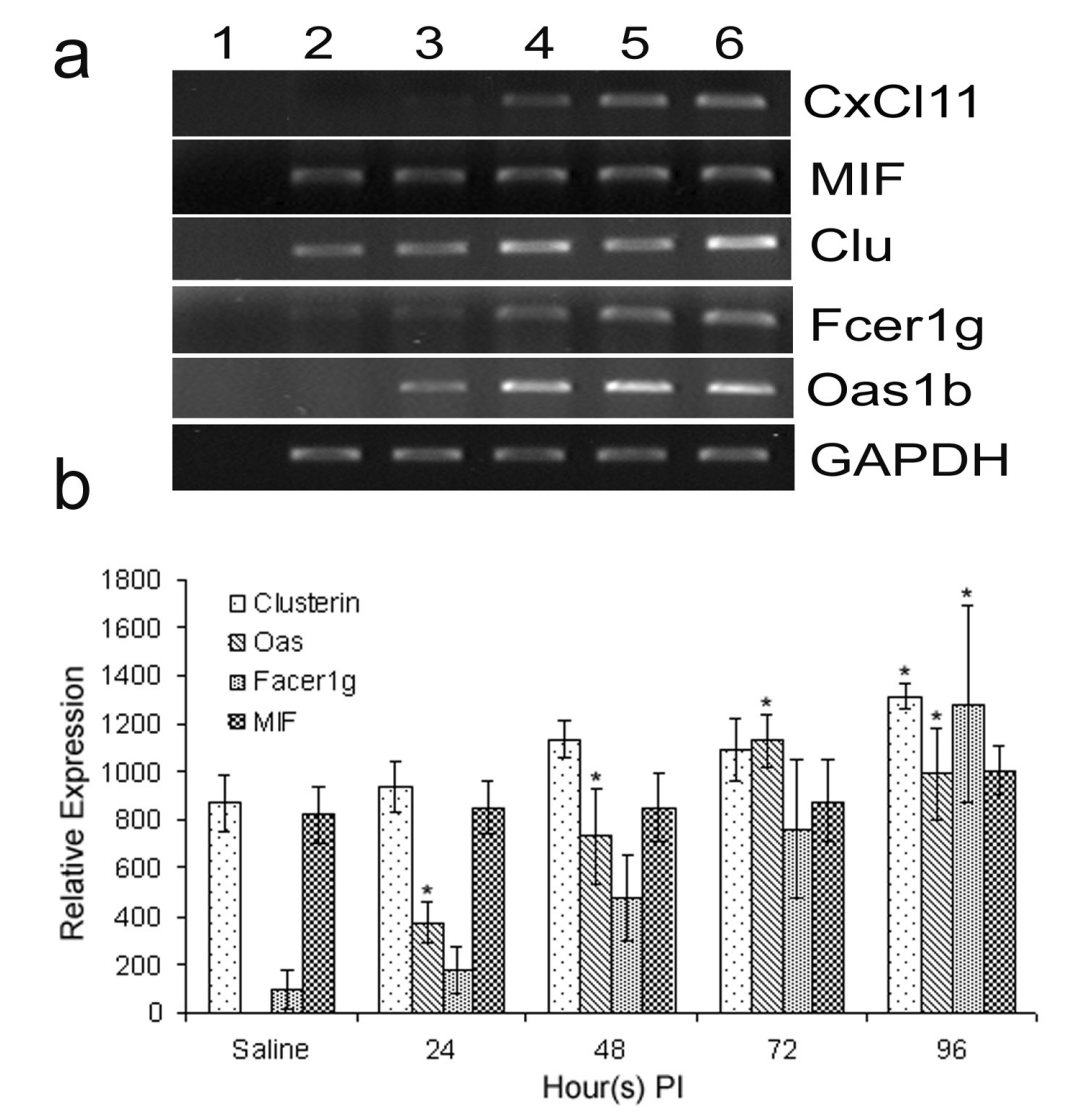
**Confirmation of selected gene expression by RT-PCR**. (a) RT-PCR was performed for *Oas1b*, *Fcre1g*, *Clu *and *Mif genes*. Expression was normalized with housekeeping gene *GAPDH*. Lane 1: Negative control, Lane 2: uninfected saline control, Lane 3: 24 hr, Lane 4: 48 hr, Lane 5: 72 hr, Lane 6: 96 hr post infection. (b) Quantitative estimation was done by densitometry analysis of PCR product using Scion Image Analysis Software (Scion Corporation, Frederick, Maryland 21701). Values were normalized to the corresponding values of the house keeping genes for individual samples. Values are expressed as mean ± SEM. * p ≤ 0.05 compared to uninfected control.

Randomly selected genes identified in the microarrays were analyzed by semi-quantitative RT-PCR analysis in 3–4 biological replicates. Specific amplification was confirmed by sequencing PCR products as described in materials and methods (data not shown). As shown in Fig 4, the expression of genes *Oas1b*, *Fcre1g *and *Clu *was up-regulated in mouse brain infected with VEEV. Although RT-PCR analysis was not quantitative, consistent with microarray analysis, gene expression of *Fcer1g*, *Oas1b *and *Clu *was higher at 48, 72 and 96 hr post infection as compared to saline control and 24 hr post infection (Fig 4)

## Discussion

The proximal cause of death following VEEV infection is attributed to the host immune response to replicating virus resulting in lethal encephalitis [8]. Therefore, it is important to study the kinetics of changes in gene expression in the brain of the VEEV infected host to understand the resulting immune response and to identify potential therapeutic targets and markers for treatment of VEEV infection. In the present study, total RNA isolated from mouse brain was used. This consisted of RNA from all the cell types present in the VEEV infected mouse brain. The goal of the study was to characterize the overall molecular frame work of gene expression in the brain. In this way, the changes in gene expression could be directly correlated with the tissue pathology. Since different cell types, individually, may respond differently to VEEV infection it is imperative to study the overall gene expression patterns during disease progression in tissue containing all the relevant cells.

Consistent with the histopathological changes, microarray analysis showed over-expression of the genes involved in the various immune responses towards the pathogen. For example *Oas1b, Ifi27, Ifih1 and Irf7 *genes were up-regulated at 72 and 96 hr post infection. Elevated level of *Oas1b *has been shown to inhibit West Nile virus (WNV) replication by reducing the positive strand viral RNA level in cells [14]. Since VEEV is a positive single strand RNA virus, increased *Oas1b *levels may also potentially inhibit VEEV replication. The anti-VEEV activity of interferon (IFN) and interferon regulatory factor (IRF) is also well established [15,16]. *IFN α/β *knockout mice showed increased and early spread of VEEV into brain, and *IRF2 *knockout mice showed increased susceptibility to otherwise avirulent strains of VEEV [17].

The ability of microglia, the brain's resident macrophage, to present antigen associated with MHC class II to T cells, allows these normally quiescent cells to play a critical role in shaping the outcome of many neurological diseases [18,19]. Activated glial cells are observed in the brains of VEEV-infected mice [10,11]. Consistent with these reported observations, upregulated MHC class II loci genes such as *H2-D1, H2-D4 H2-Q7 and H2-T23 *in the present study may reflect the activated state of glial cells in the brain of VEEV infected mice. Several molecules associated with MHC-I-mediated presentation such as *B2m, Fcerg1 and Cd1d1 *were also upregulated in our study. Beta 2 microglobulin (*B2m*) was also upregulated in acquired immunodeficiency syndrome encephalitis and JC virus infection of the CNS [20,21]. Fc-receptors are important in antigen processing/presentation of myelin proteins during the autoimmune response in the CNS [22] and contribute to inflammatory damage in the CNS. *Cd1d1 *plays a critical role in the regulation of cytokine production after an acute virus infection [23]. Thus, in the current study, VEEV infection in mice induces a similar host immune response as observed with other viruses.

VEEV infection of the CNS has been shown to result in neurodegeneration, perivascular cuffing with infiltrating lymphocytes, gliosis, cerebral edema and apoptotic neurons associated with astrogliosis in the regions of the brain which is free of VEEV antigen [10]. Therefore, it is not clear whether VEEV is directly or indirectly involved with these changes. To understand this phenomenon we investigated whether the expression of chemokines, cytokines and other inflammatory genes is altered in VEEV infected brain. Interestingly, chemokines such as *Cxcl9, Cxcl10, Cxcl11, Cxcl13, Ccl3, Ccl5 *and *Ccl12*, Fc-receptor such as *Fcer1g*, and *Mif *genes were upregulated. These chemokines may contribute to the influx of neutrophils and lymphocytes as observed in the brain (Fig 2) and the severity of the encephalitis during VEEV infection. Fc-receptor,*Fcerg1 *may contribute in VEEV antigen presentation to T cells and induction of inflammatory cytokines. The cytokine expression at the site of inflammation is suggested to be the outcome of interaction of glucocorticoids and macrophage migration inhibitory factor (Mif) [24-26]. Though over-expressed in the microarray, only a marginal increase in expression of *Mif *was observed at 96 hr post infection by PCR, where increases in inflammation and neutrophil migration were also observed.

Several apoptosis related genes were also upregulated.*Clusterin (Clu)*, an anti-apoptotic factor, activates microglia to secrete neurotoxic agents [27]. *Clu*-activated microglia showed increased MHC class II expression, secreted reactive nitrogen intermediates and *TNF-α *[27]. VEEV infection of the brain also results in the activation of microglia [11,17] and thus *Clu *may contribute to the gliosis observed in VEEV pathogenesis. The caspase recruitment domain (CARD) is a protein-binding module that mediates the assembly of CARD-containing proteins into apoptosis and NFκB signaling complexes [28]. CARD14, a membrane-associated guanylate kinase (MAGUK) family member containing CARD has been implicated in the antigen-specific signaling by the TCR complex via BCL10-mediated NFκB activation [29]. In our study, *CARD14 *upregulation was concomitant with the increased inflammation and enhancement in apoptotic neurons. CARD14 may be involved in the VEEV antigen-initiated signaling in lymphocytes or glial cells and/or in the apoptotic pathways in neurons. Faim2 inhibits Fas-mediated apoptosis and helps in protecting foreign antigen-specific B cells during potentially hazardous interactions with FasL-bearing T cells [30]. Faim2 was upregulated following VEEV infection and may be involved in the activation and survival of immune cells in the brain. The paradoxical findings on the over-expression of pro-apoptotic CARD 14 and anti-apoptotic Faim2 may be due to their expression by different cell population in the brain.

## Conclusion

Our results show a complex immune response to VEEV infection. Several pathways seem to interact for the final outcome of the disease. Thus, suppression of the inflammatory response and enhancement of the antiviral pathways may help in reducing the severity of the disease. We have identified several genes *e.g*., chemokines, *Oas1b, Fcerg1, Mif *and *Clu *which may provide potential targets for therapy against VEEV infection in the future.

## Methods

### Animals

Six to ten week old male CD-1 mice were obtained from Charles River Laboratories, Wilmington, MA. Mice were housed in micro isolator cages and were provided food and water ad libitum. A 12 hr light/dark cycle was maintained. All experiments were carried out in a bio-safety level 3 (BSL-3) facility and in accordance with the Guide for the Care and Use of Laboratory Animals (Committee on Care And Use of Laboratory Animals of The Institute of Laboratory Animal Resources, National Research Council, NIH Publication No. 86–23, revised 1996).

### Virus and Challenge Procedure

A full length cDNA clone of VEEV subtype IA/B, V3000 [5] was used in the present study. Stock virus suspension was diluted in 1× Dulbecco's Phosphate Buffered Saline (DPBS) (Gibco BRL, Invitrogen Corporation Carlsbad, CA) supplemented with 0.1% fetal bovine serum (FBS) to 1,000 plaque forming units (pfu)/25 μl. Mice were anesthetized lightly using inhalation anesthesia, isoflurane and 1000 pfu of V3000 in 25 μl volume was injected in the left rear footpad. Control animals were injected with 25 μl of 1× DPBS supplemented with 0.1% FBS.

### Histopathology and Immunohistochemistry (IHC) for VEEV antigen

Two animals from each group (n = 10) were anesthetized using isoflurane and sacrificed at 24, 48, 72 and 96 hr post infection (pi), control mice were sacrificed at 96 hr pi. Brains were collected and the right brain hemisphere was fixed in 10% buffered neutral formalin (BNF) for 3–4 weeks. The left brain hemisphere was snap frozen at -80°C. Tissues were then transferred into fresh BNF, routinely processed, and embedded in paraffin sections. Immunostaining was performed using rabbit polyclonal antiserum raised against VEEV (kindly provided by Cindy Rossi and Dr George Ludwig, USAMRIID) by an indirect avidin-biotin-immunoperoxidase technique (Vectastain ABC Elite, Vector laboratories, Burlingame, CA) as described before [31]. Briefly, sections were placed on poly-L-lysine coated slides, deparaffinized and hydrated. Endogenous peroxidase activity was blocked with 3% hydrogen peroxide in methanol for 10 min. Non-specific staining was blocked with 2.5% normal horse serum. Sections were incubated overnight with rabbit polyclonal antiserum against VEE virus (1:10000) at 4°C. To ascertain that the reaction of antibody was specific, sections from each test were incubated with normal serum IgG separately. Slides were washed with phosphate-buffered saline (PBS). Biotinylated secondary antibody IgG (H+L) was added and incubated for 30 min. Sections were incubated with streptaavidin-peroxidase complex for 10 min and stained with diaminobenzidine (DAB) (Peroxidase substrate kit DAB, Vector laboratories, Burlingame, CA), counter stain used was Harris hematoxylin.

### Isolation of total RNA

Frozen brain tissues were minced over ice and transferred to 1.5 ml microfuge tubes. Total RNA was extracted using TriZol kit (Invitrogen life technologies, Carlsbad, CA) and quantitated spectrophotometrically using Beckman DU640 Spectrophotometer (Beckman instruments Inc., Columbia, MD, USA). RNA quality was determined electrophoretically on 1% agarose formaldehyde gel.

### Microarray studies

High quality oligonucleotide glass arrays were produced containing a total of 16,463 seventy-mer oligonucleotides chosen from 750 bases of the 3' end of each ORF (open reading frame) (Operon Inc. Valencia, CA). These 16,463 oligonucleotides represent 15,000 genes. The high quality microarrays were produced in house (at CBER microarray laboratory) by spotting 70-mer oligonucleotides on poly-L-lysine coated glass slides by Gene Machines robotics (Omnigrid, San Carlos, CA). The quality of printed arrays was confirmed as described [32]. Only high quality arrays that passed our quality control tests were used for these experiments. We have followed the MIAME (minimum information about a microarray experiment) guidelines for the presentation of our data [33]. Microarray was performed as described earlier [34]. Various steps involved in the microarray are as follows:

i) Probe preparation: Labeled cDNA probes were produced as described [35,36]. Briefly, 5 μg of total RNA was dissolved in 12 μl of DEPC water and incubated at 70°C for 5 minutes along with 1 μl of aminoallyl-oligo dT (5' amino-modified) primer and quickly chilled for 3 minutes. Then, 2 μl 10× first strand buffer, 1.5 μl Stratascript RT enzyme (Stratagene, La Jolla, CA), 1.5 μl 20× aminoallyl dUTP (mixture of 100 mM dATP, dGTP, dCTP, dTTP from Invitrogen, Carlsbad, CA and 50 mM aminoallyl dUTP from Ambion, Austin, TX) and 2 μl of 0.1 M dithiothreitol (DTT, Invitrogen, Carlsbad, CA) were added and incubated for 90 minutes at 42°C. After incubation, volume of the reaction mixture was raised to 60 μl with 40 μl of DEPC water.

cDNA was purified by MinElute column (Qiagen, Valencia, CA). 300 μl of binding buffer PB was added to the coupled cDNA and mixed thoroughly. The mixture was applied to the MinElute column, and centrifuged for 1 min at max speed. After discharging the flow-through, 600 μl of washing buffer PE was added to the column, and centrifuged for 1 min at max speed. The flow-through was discharged and the washing repeated. The columns were then placed into a fresh eppendorf tube and 10 μl elution buffer added to the center of the membrane, incubated for 1 min at room temperature, centrifuged for 1 min at max speed and probes were collected. Finally, 3 μl of 2× coupling buffer (1 M NaHCO3, pH 9.0) and 5 μl Cy3 and 5 μl Cy5 (Amersham Biosciences, UK) dye mixed into eluted buffers derived from both saline and VEE infected samples respectively and incubated at room temperature in dark for 90 minutes. Saline control probes were labeled with Cy3 and probe from VEEV infected samples were labeled with Cy5. After incubation, the volume was raised to 60 μl by DEPC water and then cDNA was purified by MinElute column and eluted with 13 μl elution buffer by centrifugation.

ii) Hybridization: For hybridization, 36 μl hybridization mixture [26 μl cDNA mixture, 1 μl (10 μg) COT-1 DNA, 1 μl (8–10 μg) poly(dA), 1 μl yeast tRNA (4 μg), 6 μl 20× SSC and 1 μl 10% SDS] was pre-heated at 100°C for 2 minutes and cooled for 1 minute. Total volume of probe was added on the array and covered with cover slip (22 mm × 40 mm). Slides were placed in hybridization chamber (Genemachines) and 20 μl water was added to far end of slide (to maintain humidity), and incubated overnight (10–16 hr) at 65°C. Slides were then washed for 2 minutes each into 2× SSC, 1× SSC and 0.1× SSC and spin-dried.

iii) Data filtration, normalization, and analysis: Microarray slides were scanned in both Cy3 (532 nm) and Cy5 (635 nm) channels using Axon GenePix 4000B scanner (Axon Instruments, Inc., Foster City, CA) with a 10-micron resolution. Scanned microarray images were exported as TIFF files to GenePix Pro 5.1 software for image analysis. The raw images were collected at 16-bit/pixel resolutions with 0 to 65,535 count dynamic range. The area surrounding each spot image was used to calculate a local background and subtracted from each spot before Cy5:Cy3 ratio calculation. The average of the resulting total Cy3 and Cy5 signal gave a ratio that was used to normalize the signals. Each microarray experiment was globally normalized to make the median value of the log2-ratio equal to zero. The normalization process corrects for dye bias, PMT (Photo multiplier tube) voltage imbalance, and variations between channels in the amounts of the labeled cDNA probes hybridized. The data files representing the differentially expressed genes were then created.

For advanced data analysis, data files (in gpr format) and image (in jpeg format) were imported into mAdb (microarray database, Center for Information Technology, National Cancer Institute, Bethesda, MD, USA), and normalized by software tools provided by National Institutes Health, Center for Information Technology. Spots with ≥1.5 fold higher expression with at least 150-fluorescence intensity in either channel or 30 μm spot size were considered as good quality spots for analysis with additional filtration. These advanced filters prevented the potential effect of the poor quality spots in data analysis. All VEEV infected samples from four different time points were hybridized in duplicates (biological replicate). The data were further analyzed by Gene Ontology for Functional Analysis (GOFFA) Library of Arraytrack software [36].

### Reverse Transcription (RT) and Polymerase Chain Reaction (PCR)

cDNA was synthesized using the Superscript first strand synthesis system for RT-PCR kit (Invitrogen Inc. Carlsbad, CA). Briefly, primer mix (1ugRNA, dNTP, oligo dts) was incubated at 65°C for 5 min then mixed with reaction mixture (10× PCR buffer, 25 mM MgCl_2_, 0.1 M DTT, RNase inhibitor) and incubated at 42°C for 2 min. cDNA synthesis was done using RT enzyme (SSII) at 42°C for 50 min. The reaction was stopped by incubating at 70°C for 15 min. Residual RNA was digested by E. *coli *RNase H at 37°C for 20 min and samples were stored at -20°C. PCR was performed to validate few of the genes that were identified in the microarray. Primers and conditions used for the different genes are as follows. Clu: forward primer (BC075668.1: 36–52) 5'GACTCCAGATTCCAAGG'3, reverse primer (BC075668.1: 419-401) 5'GGTATGCTTCAGGCAGGGC'3 (95°C/30 s, 50°C/45 s, 72°C/45 s: 20 cycles),; Oas1b: forward primer (BC012877.1: 250–266) 5'GCTCAAGGGCAGGTCAG'3, reverse primer (BC012877.1: 652-635) 5'GGTTGGGCGACAGTTCAG'3 (95°C/30 s, 52°C/45 s, 72°C/45 s: 22 cycles); Mif: forward primer (NM_010798.2: 19–37) 5'CTGGCTTGGGTCACACCGC'3, reverse primer (NM_010798.2: 383-367) 5'CGTAATAGTTGATGTAG'3 (95°C/30 s, 45°C/45 s, 72°C/45 s: 23 cycles); Fcer1g: forward primer (NM_010185.4: 207–225) 5'TATGGTATTGTCCTTAC'3, reverse primer (NM_010185.4: 429–412) 5'CCAAGAGGGCTCGGAGAG'3 (95°C/30 s, 49°C/45 s, 72°C/45 s: 23 cycles); CxCl11 primer mix was purchased from Superarray Bioscience Corporation, Frederic MD, RefSeq Accession # NM_019494.1, position 693–713 (95°C/30 s, 55°C/45 s, 72°C/45 s: 23 cycles); GAPDH: forward primer 5'CCATCACCATCTTCCAGGAGCGAG'3, reverse primer 5'CACAGTCTTCTGGGTGGCAGTGAT'3 (95°C/30 s, 52°C/45 s, 72°C/45 s: 25 cycles). PCR products were visualized by electrophoreses over 1.2% agarose gel and staining with ethidium bromide. Specific amplification was determined by comparing the product size on gel relative to known DNA molecular weight marker. Further PCR products were sequenced and checked for specific amplification by blasting the sequence in the NCBI genome database. Briefly, PCR products were pooled from 72 and 96 hr for each sample and purified using spin QIAprep Spin Miniprep kit (Qiagen Inc. USA, Valencia, CA). Sequencing reaction was done using BigDye Version 2.1(Applied Biosystems, Foster City, CA). Product was then purified using Performa^® ^DTR Gel Filtration Cartridges (Edge BioSystems, Gaithersburg, MD). Sequencing was done in in-house facility on DNA Sequencer 3100 (Applied Biosystems, Foster City, CA).

## Abbreviations

VEEV: Venezuelan equine encephalitis virus; CNS: Central nervous system; MIAME: Minimum information about a microarray experiment; IHC: Immunohistochemistry; pfu: Plaque forming unit; H&E: Hematoxilin and Eosin; MST: Mean survival time; TCR: T-cell receptor; BBB: Blood brain barrier; AP: Antigen presentation.

## Authors' contributions

AS conducted the mouse experiments, IHC, histology, RNA isolation, RT-PCR, sequencing and drafted the manuscript. BB carried out microarrays and initial data analysis using different software and participated in drafting of the manuscript. AS and BB participated in microarray analysis. RKP and RKM conceived of the study, and participated in its design and coordination and helped to draft the manuscript. All authors read and approved the final manuscript.

## Supplementary Material

Additional file 1 **Supplementary table-1**: *Down modulated genes in VEEV infected mice brain*. Values are expressed as mean ± SEM. "-" indicates not expressed in biological replicates. Click here for file
