# Supplementary material for: Venezuelan equine encephalitis virus infection causes modulation of inflammatory and immune response genes in mouse brain
Source: BMC Genomics. 2008 Jun 16;9:289. doi: 10.1186/1471-2164-9-289 (PMC2440554; doi:10.1186/1471-2164-9-289)
Supplement: Additional file 1 — Supplementary table-1: Down modulated genes in VEEV infected mice brain. Values are expressed as mean ± SEM. "-" indicates not expressed in biological replicates. [file 1471-2164-9-289-S1.doc]

| **UniGene ID** | **Gene(s)** | **Expression (Hours post infection)** | | | |
| --- | --- | --- | --- | --- | --- |
|  |  | **24** | **48** | **72** | **96** |
| Mm.335292 | Actr6 (ARP6 actin-related protein 6 homolog (yeast)) | 0.349 ±0.02 | 0.604 ±0.12 | 0.671 ±0.19 | 0.497 ±0.10 |
| Mm.415865 | Ndufa4 (NADH dehydrogenase (ubiquinone) 1 alpha subcomplex, 4) | 0.327 ±0.01 | 0.503 ±0.09 | 0.707 ±0.23 | 0.384 ±0.13 |
| Mm.5246 | Ppia (peptidylprolyl isomerase A) | 0.525 ± 0.007 | 0.484 ±0.08 | 1.177 ±0.33 | 0.682 ±0.26 |
| Mm.21529 | Rpl37a (ribosomal protein L37a) | 0.567 ±0.004 | 0.502 ±0.11 | 1.403 ±0.17 | 0.801 ±0.38 |
| Mm.268797 | Rps12 (ribosomal protein S12) | 0.597 ±0.005 | 0.477 ±0.09 | 1.495 ±0.09 | 0.781 ±0.43 |
| Mm.378898 | Cox7c (cytochrome c oxidase, subunit VIIc) | 0.384 ±0.01 | 0.497 ±0.11 | 0.869 ±0.14 | 0.437 ±0.19 |
| Mm.2319 | Stmn3 (stathmin-like 3) | 0.607 ±0.005 | 0.483 ±0.10 | 1.245 ±0.38 | 0.742 ±0.26 |
| Mm.318 | 2010107E04Rik (RIKEN cDNA 2010107E04 gene) | 0.499 ±0.06 | 0.473 ±0.10 | 1.015 ±0.35 | 0.601 ±0.27 |
| Mm.30478 | Rpl39 (ribosomal protein L39) | 0.600 ±0.02 | 0.355 ±0.01 | 1.349 ±0.42 | 0.769 ±0.42 |
| Mm.358632 | Rpl30 (ribosomal protein L30) | 0.495 ±0.003 | 0.470 ±0.14 | 1.348 ±0.24 | 0.754 ±0.37 |
| Mm.377071 | Mrps33 (mitochondrial ribosomal protein S33) | 0.348 ±0.005 | 0.411 ±0.08 | 0.855 ±0.16 | 0.453 ±0.18 |
| Mm.261679 | Rps26 (ribosomal protein S26) | 0.639 ±0.02 | 0.351 ±0.04 | 1.652 ±0.15 | 0.760 ±0.41 |
| Mm.2766 | Pvalb (parvalbumin) | 0.383 ±0.02 | 0.564 ±0.26 | 0.536 ±0.37 | 0.376 ±0.02 |
| Mm.5023 | Pcp4 (Purkinje cell protein 4) | 0.529 ±0.01 | 0.325 ±0.04 | 1.461 ±0.17 | 0.516 ±0.16 |
| Mm.379061 | Timm8b (translocase of inner mitochondrial membrane 8 homolog b) | 0.272 ±0.03 | 0.387 ±0.10 | 0.805 ±0.17 | 0.405 ±0.20 |
| Mm.29722 | Usmg5 (upregulated during skeletal muscle growth 5) | 0.333 ±0.015 | 0.335 ±0.05 | 0.813 ±0.25 | 0.495 ±0.22 |
| Mm.3229 | Rpl26 (ribosomal protein L26) | 0.507 ±0.007 | 0.369 ±0.09 | 1.316 ±0.29 | 0.614 ±0.40 |
| Mm.133551 | Atp5j2 (ATP synthase, H+ transporting, mitochondrial F0 complex, subunit f, isoform 2) | 0.443 ±0.02 | 0.340 ±0.08 | 0.735 ±0.11 | 0.437 ±0.17 |
| Mm.219685 | Pigf (phosphatidylinositol glycan anchor biosynthesis, class F) | 0.345 ±0.02 | 0.6538/ - | 0.700 ±0.07 | 0.526 ±0.01 |
| Mm.331007 | Ndufc1 (NADH dehydrogenase ubiquinone) 1, subcomplex unknown, 1) | 0.482 ±0.03 | 0.562/ - | 0.836 ±0.19 | 0.469 ±0.09 |
| Mm.12481 | Cetn3 (centrin 3) | 0.254 ±0.009 | 0.586/ - | 0.649 ±0.07 | 0.457 ±0.08 |
| Mm.24848 | Deb1 (differentially expressed in B16F10 1) | 0.369 ±0.04 | 0.530/ - | 0.807 ±0.19 | 0.408 ±0.13 |
| Mm.249110 | Snrpe (small nuclear ribonucleoprotein E) | 0.419 ±0.001 | 0.643/ - | 0.701 ±0.09 | 0.477 ±0.17 |

**Supplementary Table 1**: Down modulated genes in VEEV infected mice brain.

Values are expressed as mean ± SEM. "-" indicates not expressed in biological replicates.
